# An integrated analysis of hypoxic–ischemic encephalopathy‐related cell sequencing outcomes via genes network construction

**DOI:** 10.1002/ibra.12025

**Published:** 2022-03-15

**Authors:** Hong‐Su Zhou, Ting‐Bao Chen

**Affiliations:** ^1^ Department of Laboratory Zoology Kunming Medical University Kunming Yunnan China

**Keywords:** bioinformatics, hypoxic–ischemic encephalopathy, JAK‐STAT signaling pathway, single‐cell sequencing, STAT

## Abstract

Hypoxic–ischemic encephalopathy (HIE) is one of the main causes of morbidity and severe neurological deficits in neonates. This study aimed to find core genes and their potential roles in HIE with the help of single‐cell sequencing (SCS) technology and genes network construction. We collected and screened an HIE genes data set from the Pubmed database to analyze differential expression, and the differential values of genes were ≥3 or ≤−3 in gene expression. We constructed a protein–protein interaction (PPI) network by the string, which was also verified by Cytoscape 3.8.2. Functional enrichment analysis was performed to determine the characteristics and pathways of the core genes. We examined two meaningful papers and integrated all genes by SCS, which were classified into 12,093 genes without duplicates, 217 shared genes, and 11,876 distinct genes. Among 217 genes, the signal transducer and activator of transcription (STAT) family was the most targeted gene in the PPI network. Moreover, Gene Ontology and Kyoto encyclopedia of genes and genome analysis showed that the process in response to virus and the JAK‐STAT signaling pathway play significant roles in HIE. We also found that 54 screened genes were highly expressed, while three genes (B2M, VIM, and MRPS30) were different in the heat map and differential genes expression exhibition. VIM, as an essential portion of the brain's cytoskeleton, is closely linked to STAT and neurologic development. From the findings of SCS and bioinformatics predictive analytics model, our outcomes provided a better understanding of the roles of STAT, the JAK‐STAT signaling pathway, and VIM, which can pave an alternative avenue for further studies on HIE progression.

## Graphical abstract

Hypoxic–ischemic encephalopathy (HIE) is one of the main causes of morbidity and severe neurological deficits in neonates. We collected and screened an HIE genes data set from the Pubmed database to analyze differential expression, and the differential gene expression values of genes were ≥ 3 or ≤ −3. We constructed a protein–protein interaction (PPI) network by the string, which was also verified by Cytoscape 3.8.2. Functional enrichment analysis was performed to determine the characteristics and pathways of the core genes. We examined two meaningful papers and integrated all genes by SCS, which were classified into 12,093 genes without duplicates, 217 shared genes, and 11,876 distinct genes. Among 217 genes, the signal transducer and activator of transcription (STAT) family was the most targeted gene in the PPI network. Moreover, Gene Ontology and Kyoto encyclopedia of genes and genomes analysis showed that the process in response to virus and the JAK‐STAT signaling pathway play significant roles in HIE. We also found that 54 screened genes were highly expressed, while three genes (B2M, VIM, and MRPS30) were different in the heat map and genes expression exhibition. VIM, as an essential portion of the brain's cytoskeleton, is closely linked to STAT and neurologic development.

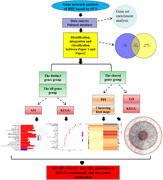

## 1 INTRODUCTION

Hypoxic–ischemic encephalopathy (HIE) is a deteriorative consequence of hypoxic–ischemic brain injury (HIBI), also one of the severe complications and diseases for neonates, causing many destructive neurological events in the perinatal period,^1^ for example, cerebral palsy, cognitive impairment, and mental deficiency, with the incidence rate being as high as 5%–8%.^2^ HIBI is regarded as a “waterfall” pathological change and is involved in the interaction of multiple factors and mechanisms. Though several experts and professional researchers have made great progress in examining and describing its pathophysiology and pathogenesis and therapies, the majority could only improve symptoms of impaired neurological function. Moreover, it places a huge economic burden on the family and society in the follow‐up treatments.^3^ What's worse, the pathological mechanism is not unknown.

Single‐cell sequencing (SCS) technology plays an important role in the development of biology by allowing direct observation of the characteristic observation of the basic units of life. SCS refers to sequencing and analysis of the genetic information carried by a single cell, aiming to obtain the nucleic acid sequence, transcriptional information, gene quality, and epigenetic expression profile information of a certain cell, and conduct integrated analysis at the molecular level, which can be used to screen the gene map.^4^ Arguably, SCS technology yields a comprehensive understanding of the pathogenesis of HIE from the perspective of nucleic acid molecules, gene quality information, and different types of cell function. Of course, there are still unsolved problems, such as bias in amplification, difficulty in manipulation, difficulty in processing sequencing data, and high costs of the technology,^5^ which may explain why it is still under development.

**Figure 1:**
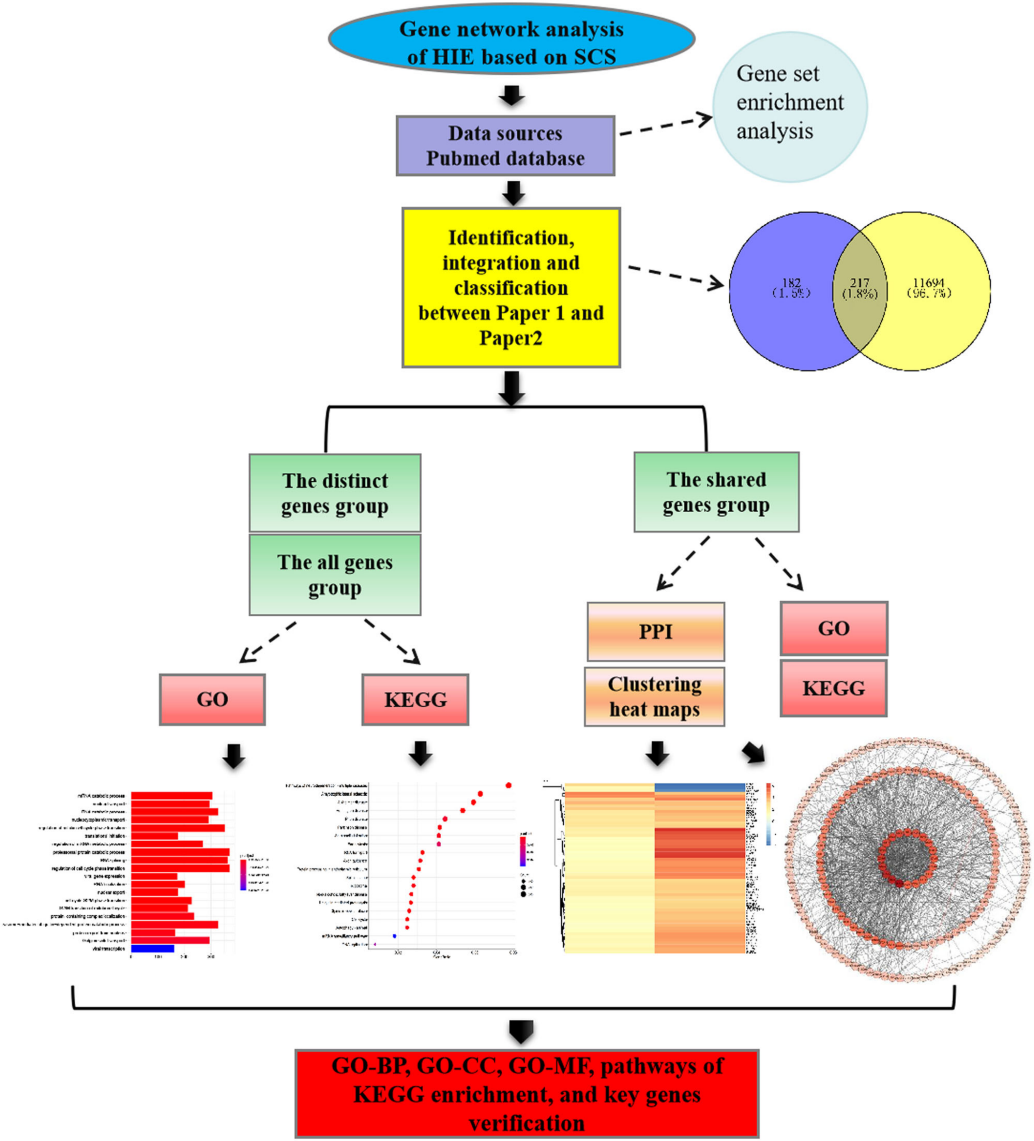
Workflow to identify key genes of hypoxic–ischemic encephalopathy. BP, biological processes; CC, cellular components; GO, Gene Ontology; KEGG, the Kyoto encyclopedia of genes and genomes; MF, molecular functions; PPI, the protein–protein interaction [Color figure can be viewed at wileyonlinelibrary.com]

Bioinformatics is a new discipline at the intersection of molecular biology and various disciplines and is covered by many fields of study. Among them, network pharmacology, which mainly investigates the effective components and potential targets of traditional Chinese medicine (TCM),^6^ is also regarded as a bioinformatics predictive analytics model for elucidating the correlation of polygenes with specific diseases. Combined with the advantages of SCS technology and the bioinformatics predictive analytics model, this article aims at searching the literature of SCS in HIE and screening out the key genes by building the genes network, which may be useful for molecular warning and treatment (the graphic abstract is shown in Figure 1).

## 2 MATERIALS AND METHODS

### 2.1 Searching for genes sequenced by SCS technology

We searched the literature on HIE with SCS (Search Strategies (“cerebral ischemia” or “cerebral hypoxia” or “cerebral ischemia and hypoxia” or “perinatal hypoxia‐ischemia or adult stroke” or “brain injury”) and (single‐cell sequencing) or (“hypoxic‐ischemic encephalopathy” or and “single‐cell sequencing”) or (neurogenesis and single‐cell sequencing) from PubMed (https://pubmed.ncbi.nlm.nih.gov/), and all data‐mined would be put into Excel to make tables for easy follow‐up.

### 2.2 Data sorting by venny graph

The genes were input into different lists with an application of the venny 2.1 database (https://bioinfogp.cnb.csic.es/tools/venny/), and automatically generated the intersection of same genes, and downloaded the pictures in the venny 2.1 database.

### 2.3 Construction and analysis of the protein–protein interaction (PPI) network

Using the string database (https://string-db.org/), the shared genes from different articles were input into the corresponding search box before setting the “Organism” option to “Homo sapiens.” We created a gene interaction map, then exported and downloaded its high‐definition interaction map and interaction relationship tables. Thereafter, this interaction map was analyzed and displayed on Cytoscape 3.7.2. The higher the score, the closer the relationship.

### 2.4 Gene ontology (GO) analysis

GO analysis is an international standardized gene functional classification system, which provides dynamically updated and controlled terms, and strictly defines concepts to comprehensively describe the characteristics of genes and their products in any organism. In this study, all targets are mapped to the gene ontology database (http://www.geneontology). In the GO item, the number of genes per term was calculated, path‐based analysis was used to characterize the biological function of genes, and R language was operated after installing RSQLite, ClusterProfiler, Org.Hs.eg, DOSE, Enrichplot, Ggplot2, Colorspace, String, and Pathview into R software version 4.0.4 (http://www.r-project.org).

### 2.5 Kyoto encyclopedia of genes and genomes (KEGG) pathway analysis

Similarly, all genes were analyzed by KEGG. Application of pathway enrichment analysis in KEGG pathway database (http://www.genome.jp/kegg/). The KEGG pathway was analyzed using R language operation. The procedure of R software version 4.0.4 installation was the same as above. The core genes were exhibited from the KEGG pathway.

### 2.6 Heat maps and differential genes expression exhibition

We screened out the data with difference in genes expression value ≥ 3 or ≤ −3. A website, https://www.omicstudio.cn/index, can visualize the heat map to compare genes' expression with each other, and use GraphPad 8.0.1 to analyze differentially expressed upregulated and downregulated genes. The change in colors represent a change in quantity, which showed the pathway influenced, and we utilized a distance matrix to analyze the clusters.

## 3 RESULTS

### 3.1 Retrieved results including the cells and expression genes from SCS

Two relevant pieces of literature were examined, whose titles were, respectively, “single‐cell transcriptomics reveals a population of dormant neural stem cells that become activated upon brain injury^7^ (hereinafter referred to as Paper 1)” and “Spatiotemporal gene expression trajectories reveal developmental hierarchies of the human cortex^8^, ^9^ (hereinafter referred to as Paper 2)”. Three hundred and ninety‐nine specific genes were obtained by sequencing a pool of adult subventricular zone NSCs in Paper 1 (Supporting Information 1 showed 399 genes). This study revealed the general pattern of NSC activation during cerebral ischemia by sequencing NSCs in the adult subventricular zone. This analysis suggested that the interferon‐γ signaling pathway induces a dormant NSC subpopulation into an initiation‐resting state, attached to downregulation of the glycolytic metabolism, and Notch and BMP signaling pathways. In Paper 2, different types of cells (including astrocytes, oligodendrocyte precursor cells, microglia, and so on) were sequenced to obtain 11,911 genes; they were in stages of peak neurogenesis in development of the human cerebral cortex (Supporting Information 2 presents all 11,911 genes; Table 1 shows different types of cells). In addition, SCS was used to explore modest transcriptional differences in robust type differences between the conversion of radial glial cascades into mature cortical neurons, revealing the expression of neurogenic transcription factors in early radial glial cells and the activation of mammalian target proteins of the rapamycin signaling pathway in lateral radial glial cells.

**Table 1:** Different types of cells sequenced by SCS

Cell names		
Astrocytes	Oligodendrocyte precursor cells	Microglia
Intermediate progenitor cells	Excitatory cortical neurons	Ventral MGE progenitors cells
Choroid plexus cells	Mural cells	Endothelial cell
Radial glia	Inhibitory cortical interneurons	Neural stem cell

Abbreviation: SCS, single‐cell sequencing; MGE, megakaryocyte.

### 3.2 The integrated three groups of genes via  the venny graph

**Figure 2:**
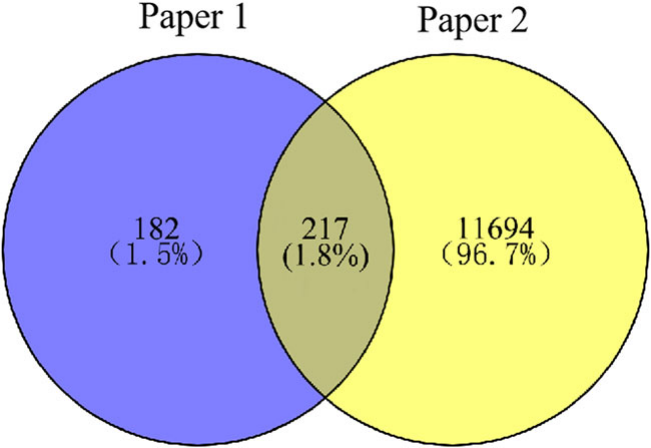
Venny diagram of the intersection of two papers [Color figure can be viewed at wileyonlinelibrary.com]

We classified all genes into three groups, including 12,093 genes removed duplicates from Paper 1 and Paper 2, 271 shared genes, 11,876 distinct genes. They been shown in Figure 2, and 271 shared genes were seen in Table 2.

**Table 2:** The shared genes list

Genes name							
A2M	CCND1	EGFL7	GPNMB	JUNB	NLRC5	RASIP1	STAB1
ADAMTSL3	CD44	EGR2	GPR158	KIT	NUPR1	RHOJ	STAT1
AHNAK	CD63	EIF2AK2	GPX1	KLF10	OAS2	RN7SK	STAT2
AKAP12	CD9	EIF3F	GRIK3	KLF4	OCLN	RNF125	STAT3
ALDH1L2	CD93	EMP1	GRM5	LBH	OSMR	RNF213	TAF4B
AMOTL1	CDKN1A	EMP3	HBEGF	LDHA	P4HA3	RPS2	TAGLN2
ANGPT1	CEBPD	ENG	HERC6	LEPR	PALMD	S100A10	TAPBP
ANGPTL4	CFH	ENO2	HMOX1	LGALS1	PARP12	S100A11	THBS1
ANXA2	CGNL1	ESAM	IER2	LGALS3	PARP14	S100A4	THBS2
APOD	CHL1	FABP5	IER3	LGALS3BP	PARP9	S100A6	TIMP1
ARHGAP28	CHURC1	FAM19A5	IFI27	LY6E	PARVB	S1PR3	TNFRSF1A
ARID5B	CLDN5	FAM46A	IFI44	LYPLA2	PDE2A	SAMD9L	TRIB1
ARPC1B	CMPK2	FAM84A	IFIH1	MADD	PER1	SDC1	TRIM25
ATF3	COL12A1	FBXO17	IFIT1	MAFF	PFKFB4	SERPING1	TRIM47
ATG3	COL16A1	FLNC	IFIT3	MASP1	PIK3R5	SERTAD1	TRIM56
ATP8A2	COL5A3	FLOT1	IFITM2	MCAT	PLK2	SHISA5	TSPO
B2M	COTL1	FLT1	IFITM3	MGAT4A	PLVAP	SLC38A5	TUBB6
BAIAP2	CP	FOLR2	IGFBP2	MRPL13	POLR2M	SLC39A14	UACA
BLOC1S3	CRELD2	FOS	IGFBP3	MRPS30	PPP1R1A	SLC40A1	UBE2L6
BRI3	CTHRC1	FOSL2	IL1R1	MRPS6	PRKAR1B	SMAD3	VIM
BST2	CTSC	GADD45A	IL33	MX1	PROS1	SNF8	VWA1
C1QB	CYR61	GADD45B	IRF1	MYC	PSMB8	SOCS3	VWF
C3	DDX58	GBP3	IRF2BPL	MYO1C	PSME1	SPRY1	XAF1
CAPG	DDX60	GFAP	ISG15	NAV3	PTPN5	SPRY4	YARS2
CAV1	DNTTIP1	GLIPR2	ISOC1	NFKBIA	PTPRB	SRGN	ZC3HAV1
CAV2	DTX3L	GLRX5	ITGA5	NFKBIZ	RAB3B	SRM	ZFP36
CCDC136	DUSP6	GPD1	ITM2A	NLRC3	RAB5A	ST8SIA5	ECM1
CCL2							

### 3.3 Construction and analysis of the PPI network

**Figure 3:**
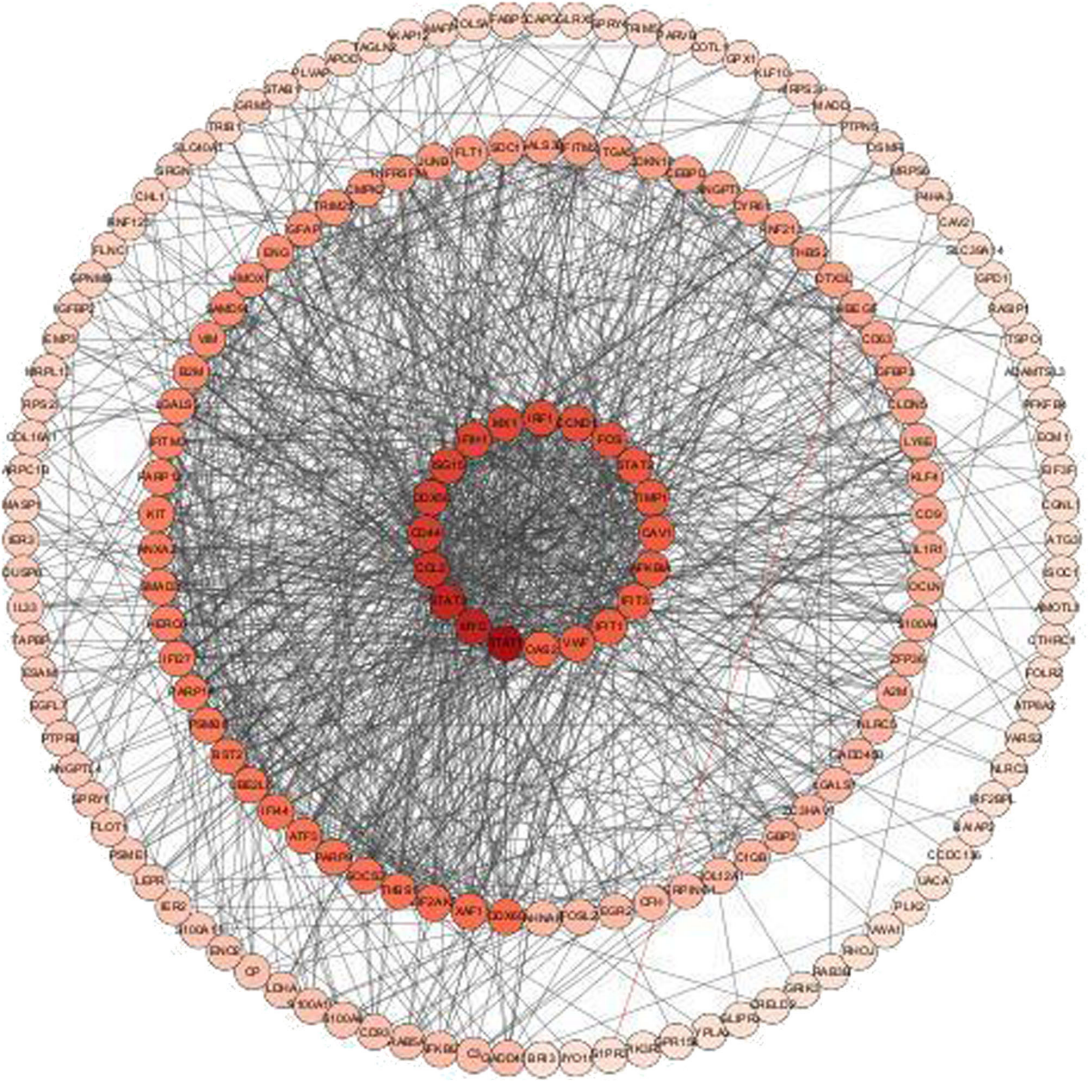
PPI relationship network. PPI, protein–protein interaction [Color figure can be viewed at wileyonlinelibrary.com]

The 271 shared genes were listed in the string (https://string-db.org/) to obtain PPI, which were imported into Cytoscape 3.8.2, and a PPI relationship network was constructed (Figure 3). This network demonstrated the interaction and crosstalk between multiple signaling pathways and genes. The signal transducer and activator of transcription (STAT) family is the most targeted gene by the shared group, including STAT1, STAT2, and STAT3.

### 3.4 GO and KEGG pathway analyses

#### 3.4.1 All the 12,093 genes

**Figure 4:**
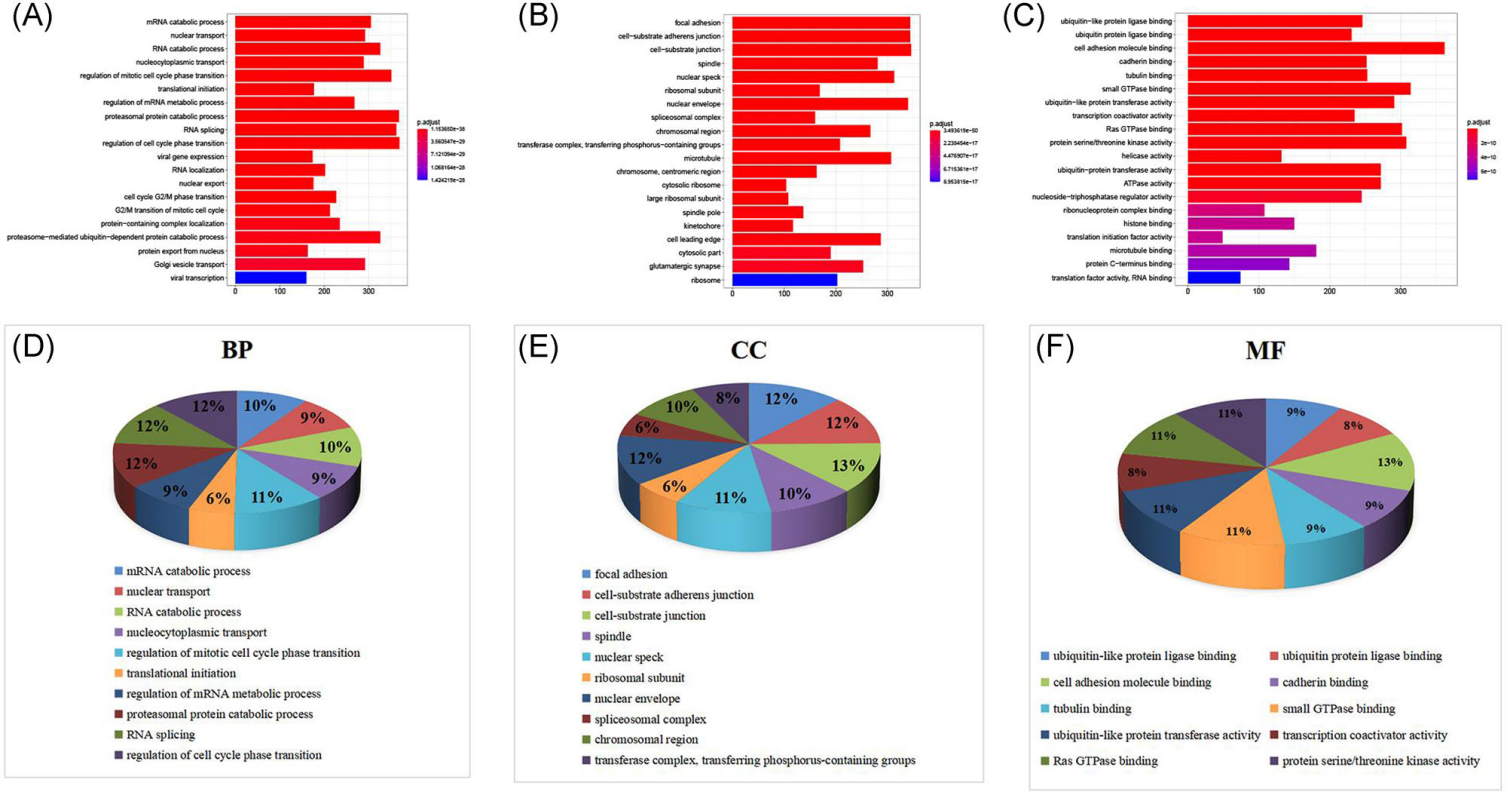
GO enrichment analysis for all genes. (A–C) Top 20 enriched components in BP, CC, and MF, respectively. The redder the color, the higher the significance. The length of the abscissa represents the number of genes. (D–F) Quantitative results of go enrichment analysis of only the top 10 in BP, CC, and MF. BP, biological process; CC, cell components; GO, Gene Ontology; MF, molecular functions [Color figure can be viewed at wileyonlinelibrary.com]

**Figure 5:**
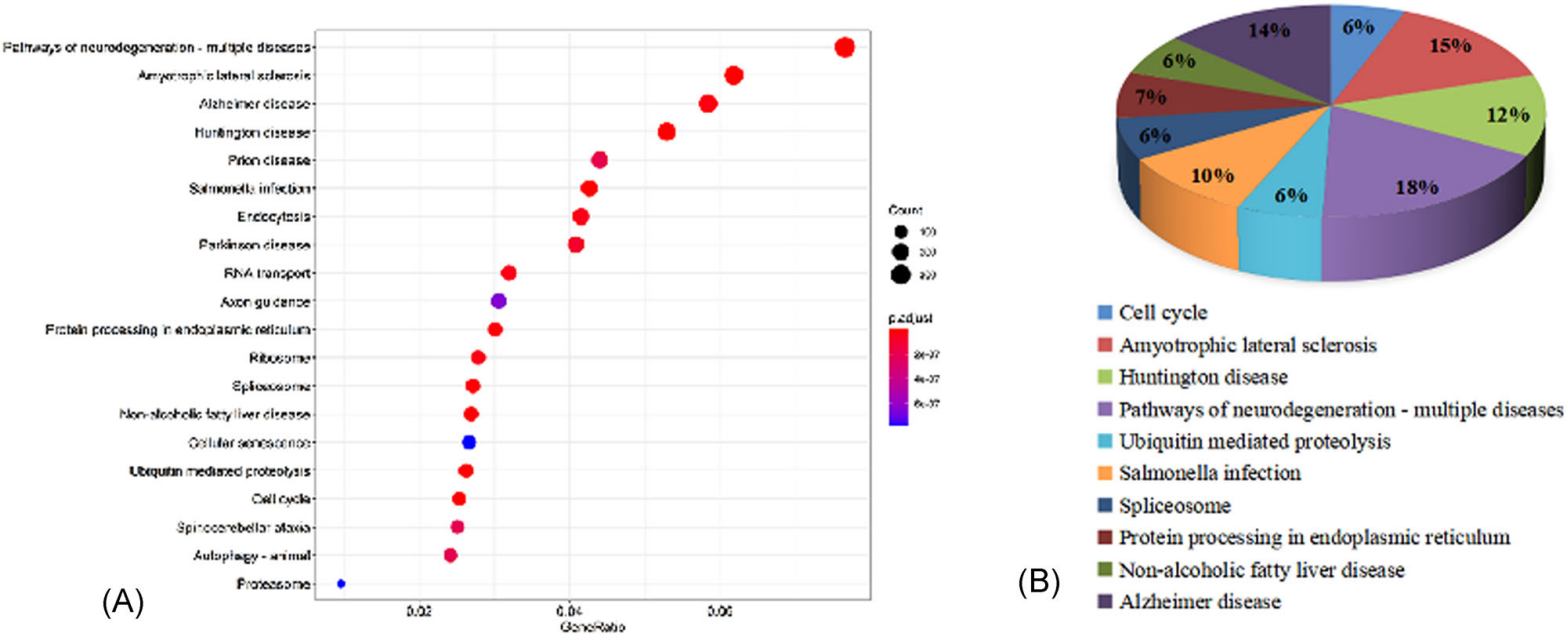
KEGG path analysis path diagram of all the genes. (A) Bubble diagram. Gene ratio refers to the ratio of enriched genes to all target genes. Counts refer to the number of enriched genes. (B) Pie chart of the top 10 pathway distributions. KEGG, Kyoto Encyclopedia of Genes and Genomes [Color figure can be viewed at wileyonlinelibrary.com]

To interpret the underlying biological roles of these genes, we subjected them to GO and KEGG enrichment analyses using the “cluster profile” package in R software. By setting the filter as adjusted *p* value < 0.05 and *q* value < 0.05, in total, 2098 GO‐biological processes (BPs), 457 GO‐cell components (CCs), 314 GO‐molecular functions (MFs), and 131 KEGG pathways were enriched. In terms of BP, the highest level of genes enrichment was the messenger RNA (mRNA) catabolic process, the focal adhesion for cell components CC, and the ubiquitin‐like protein ligase binding for MF; a chord diagram of the top 20 enriched BP, CC, and MF terms across gene lists is presented in Figure 4A–C. The top 10 items made up a pie, and were seen in Figure 4D–F. The results of the KEGG pathway analysis mainly highlighted cell cycle, amyotrophic lateral sclerosis huntington disease, and pathways of neurodegeneration‐multiple diseases (Figure 5). Additionally, the top 20 KEGG are shown in Table 3, which is compared with the shared group.

**Table 3:** The top 20 pathways enriched by target genes from the entire group

ID	Description	*p* Value	*p* Adjust	*q* Value	Count
hsa05022	Pathways of neurodegeneration—multiple diseases	1.78E−14	1.46E−12	8.08E−13	336
hsa05014	Amyotrophic lateral sclerosis	2.47E−16	4.06E−14	2.25E−14	271
hsa05010	Alzheimer's disease	6.82E−10	2.24E−08	1.24E−08	256
hsa05016	Huntington's disease	1.12E−15	1.22E−13	6.78E−14	232
hsa04151	PI3K‐Akt signaling pathway	0.000149807	0.000746767	0.000413344	225
hsa05165	Human papillomavirus infection	6.37E−06	5.66E−05	3.13E−05	218
hsa04010	MAPK signaling pathway	7.58E−08	1.19E−06	6.58E−07	203
hsa05020	Prion disease	9.09E−09	1.99E−07	1.10E−07	193
hsa05132	Salmonella infection	3.34E−12	1.83E−10	1.01E−10	187
hsa04144	Endocytosis	1.66E−09	4.20E−08	2.32E−08	182
hsa05012	Parkinson disease	4.10E−09	9.64E−08	5.33E−08	179
hsa05131	Shigellosis	1.03E−05	8.59E−05	4.75E−05	166
hsa05171	Coronavirus disease (COVID‐19)	5.23E−07	6.37E−06	3.53E−06	162
hsa04714	Thermogenesis	2.55E−05	0.000172924	9.57E−05	156
hsa04810	Regulation of actin cytoskeleton	2.70E−06	3.17E−05	1.75E−05	151
hsa05166	Human T‐cell leukemia virus 1 infection	4.08E−06	4.33E−05	2.40E−05	151
hsa05163	Human cytomegalovirus infection	7.11E−05	0.000396571	0.000219507	150
hsa04510	Focal adhesion	8.71E−08	1.30E−06	7.21E−07	145
hsa05205	Proteoglycans in cancer	5.32E−06	5.10E−05	2.82E−05	142
hsa03013	RNA transport	1.43E−09	3.93E−08	2.17E−08	140

#### 3.4.2 The 271 shared genes

**Figure 6:**
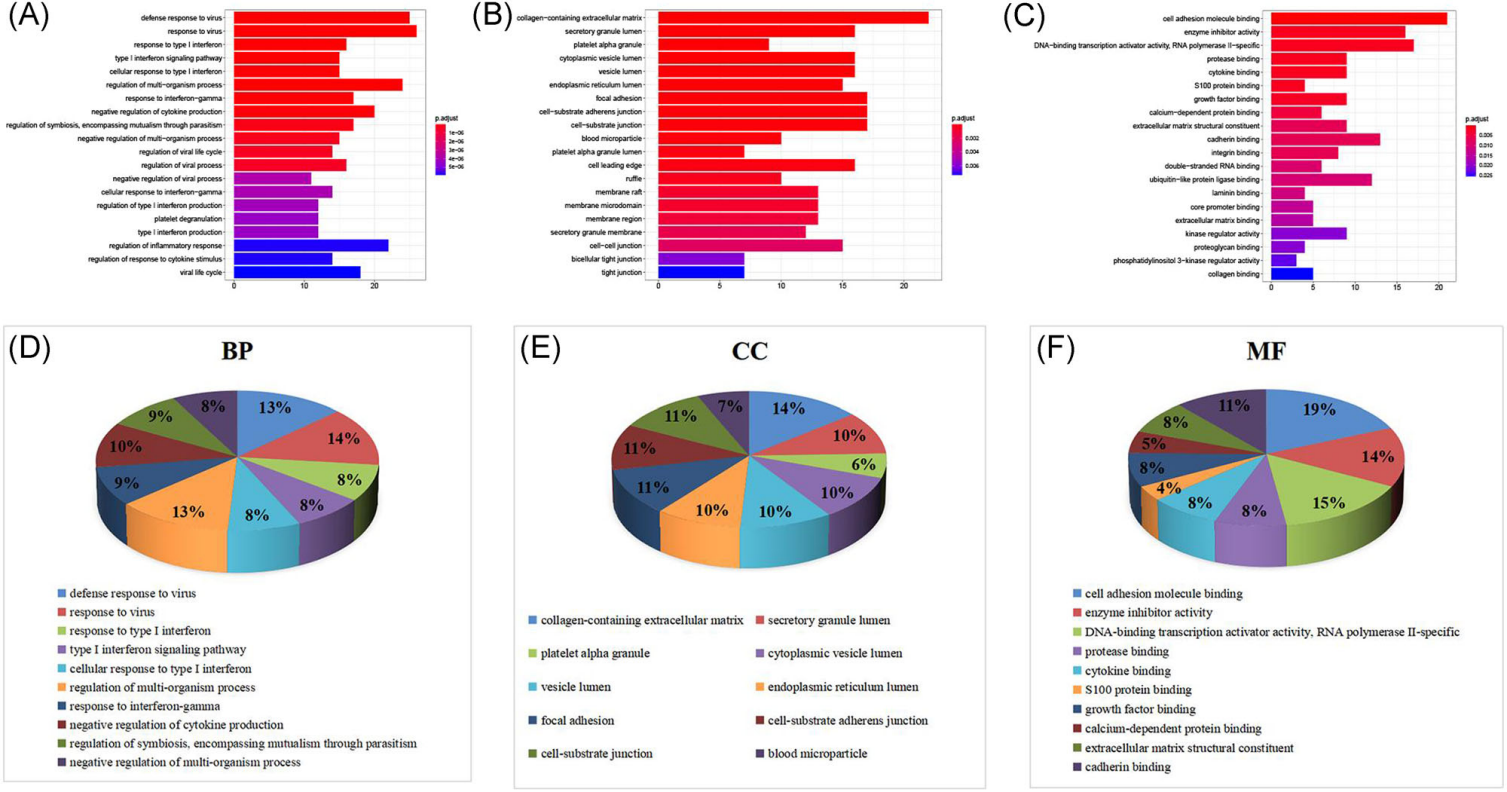
GO enrichment analysis for the shared genes. (A–C). The top 20 enriched components in BP, CC, and MF, respectively. Pathways with *p* < 0.05 were identified as significant. The length of the abscissa represents the number of genes, and color represents the *p* value. (D–F). Quantitative results of go enrichment analysis of the top 10 participated in BP, CC, and MF. BP, biological process; CC, cell components; GO, Gene Ontology; MF, molecular functions [Color figure can be viewed at wileyonlinelibrary.com]

**Figure 7:**
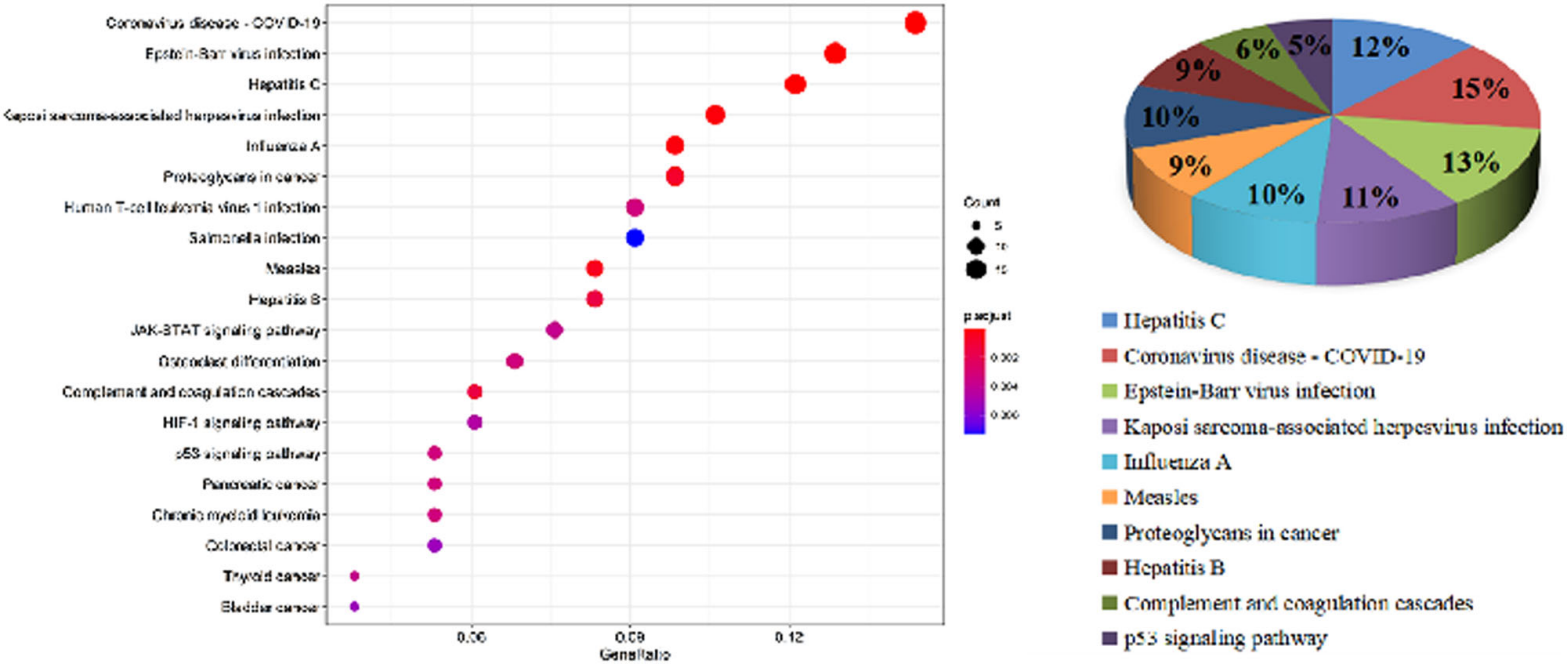
KEGG pathway of the shared genes. (A) Bubble diagram. The gene ratio refers to the ratio of enriched genes to all target genes. Counts refer to the number of enriched genes. (B) Pie chart of the top 10 pathway distributions. KEGG, Kyoto Encyclopedia of Genes And Genomes [Color figure can be viewed at wileyonlinelibrary.com]

**Figure 8:**
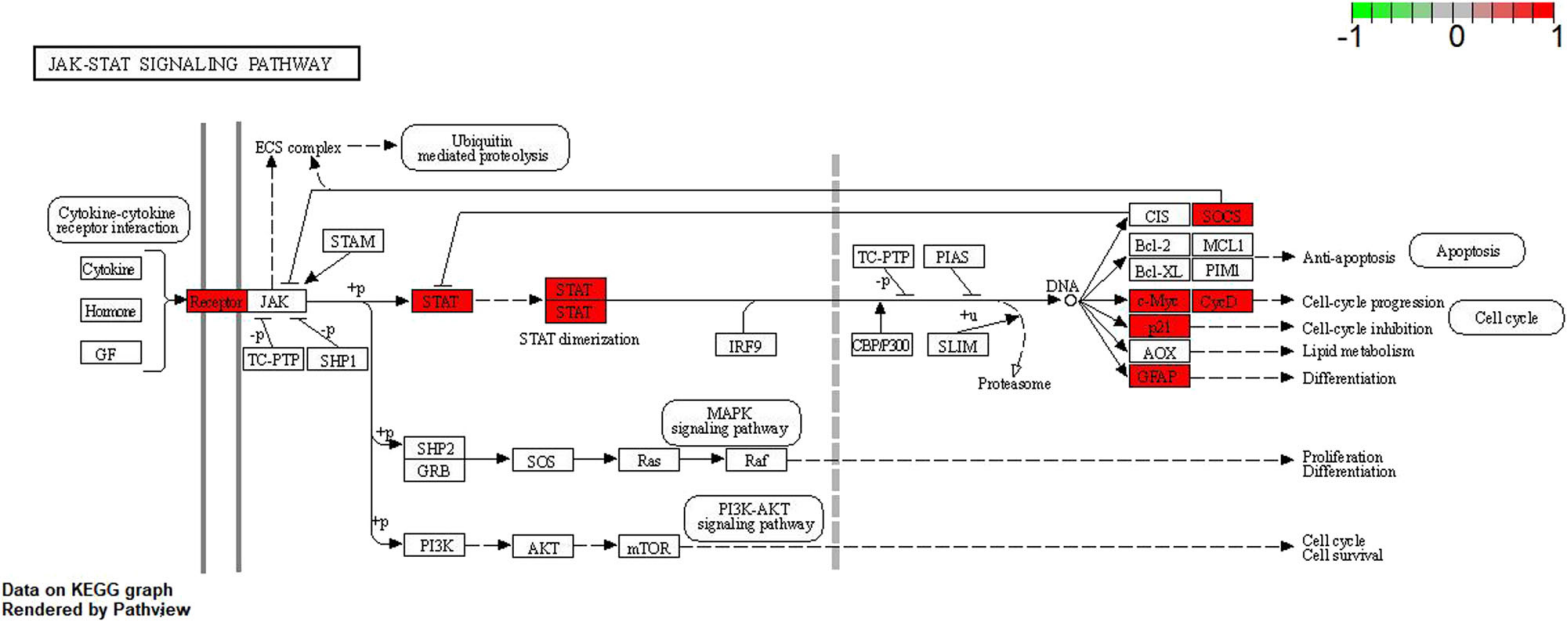
Map of the JAK‐STAT signaling pathway as the meaningful enriched pathway [Color figure can be viewed at wileyonlinelibrary.com]

Equally, in total, 485 GO‐BP, 34 GO‐CC, 25 GO‐MF, and 44 KEGG pathways were enriched, when the filter was also set as an adjusted *p* value < 0.05 and *q* value < 0.05. The defense response to the virus, collagen‐containing extracellular matrix, and cell adhesion molecules showed the highest levels of gene enrichment, respectively, for GO‐BP, GO‐CC, and GO‐MF in shared genes; a chord diagram of the top 20 enriched BP, CC, and MF terms across gene lists is presented in Figure 6A–C. For the top 10 counterparts, a pie chart was created (Figure 6D–F). The hepatitis C pathway and coronavirus disease (COVID‐19) were the highest score of KEGG pathway analysis (Figure 7). the top 20 pathways are shown in Table 4. STAT of the JAK‐STAT signaling pathway (ID: hsa04630) was the most targeted gene in Figure 3. STAT is the substrate for janus kinase (JAK)**.** The map of the JAK‐STAT signaling pathway is shown in Figure 8.

**Table 4:** The top 20 pathways enriched by target genes from the shared group

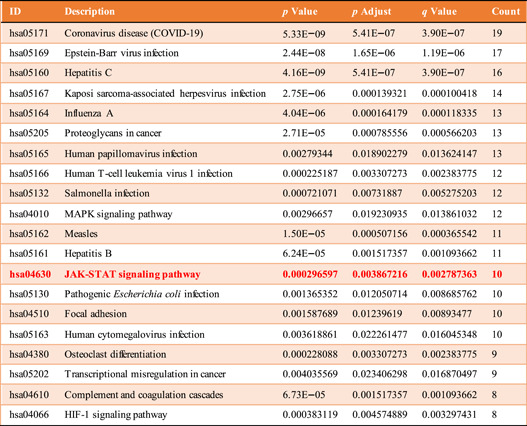

*Note*: The hsa04630 is highlighted in red due to the highest score of STAT in the PPI relationship network (Figure 3).

Abbreviations: PPI, protein–protein interaction; STAT, signal transducer and activator of transcription.

### 3.5 Up‐ and downregulation and clustering heat map of cross genes

**Figure 9:**
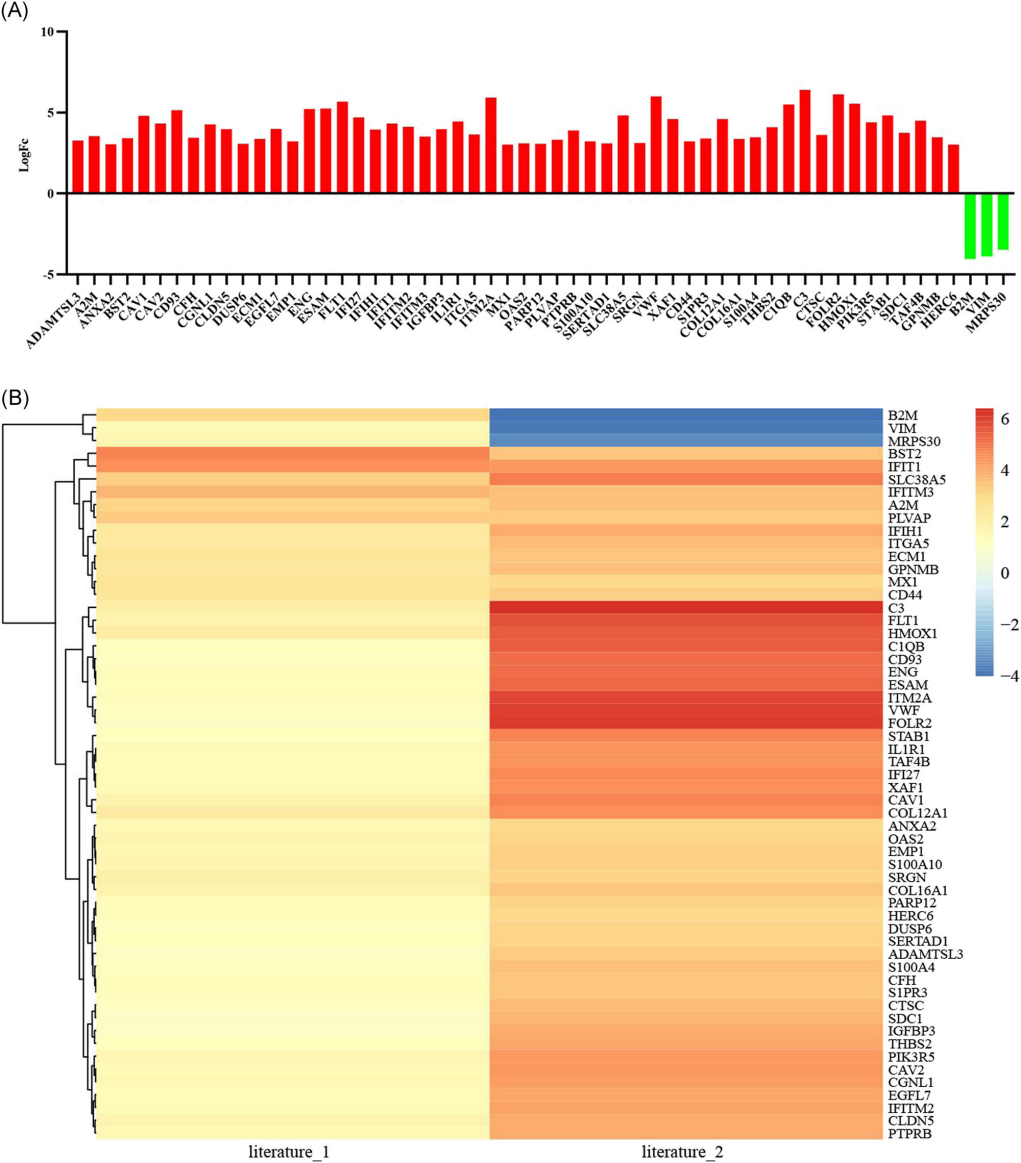
Differential gene expression analysis. (A) The bars of 54 genes represented that they were upregulated (in red), while the bars of three genes were down‐regulated (in green). (B) The main genes of two heat maps of SCS are listed in the column. These lines showed 57 sets of differentially expressed genes in Paper 1 and Paper 2, respectively. The level of upregulation parallelled saturation of warm colors; on the contrary, downregulation is highlighted in blue [Color figure can be viewed at wileyonlinelibrary.com]

In total, 57 differentially expressed genes were screened from 271 shared genes, and then, their upregulation or downregulation was determined using http://www.bioinformatics.com.cn/. Among these 57 genes, the expression of 54 genes was upregulated, and that of beta‐2 microglobulin (B2M), mitochondrial ribosomal protein S30 (MRPS 30), and Vimentin (VIM) was upregulated in Paper 1, but reversed in Paper 2 (Figure 9A,B).

## 4 DISCUSSION

This paper is based on SCS technology in the context of a scientific database that can search for two studies about sequenced cellular genomes in HIE, which were collated into three groups after integrating, including all the genes (12,093), shared genes (271), and distinct genes (11,876). PPI network displayed a group of STAT key targets out of 271 genes. GO and KEGG analyses showed that the procedures of response to virus, pathways of neurodegeneration‐multiple diseases, and the JAK‐STAT signaling pathway play significant roles in HIE. Besides, we found that changes in the expression of three genes (B2M, VIM, MRPS30) were different in two papers. Next, we elaborated on the results of GO and KEGG analyses. STAT is highlighted by an interaction between the JAK‐STAT signaling pathway and three genes.

The findings of the screened genes enrichment analysis by GO and KEGG were interesting. First, the shared genes were enriched in response to viruses (GO‐BP), collagen‐containing extracellular matrix (GO‐CC), and cell adhesion molecules (GO‐MF), which might directly impact the development and progression of HIE. This result has been validated by and consistent with previous studies. A retrospective study has shown that half of 12 infants who died from HIE died due to viral infection.^10^ Amarnath et al. showed that human papillomavirus induced neonatal leukoencephalitis.^11^ Influenza A virus could cause brain lesions including in the thalamus, bilateral caudate nuclei, subcortical white matter, and cerebellar hemispheres in neonates.^12^ In the case of GO‐CC, lack of the extracellular matrix affected the maturation of neonatal brain white matter.^13^ The induction of matrix metalloproteinases (MMPs), a class of protein hydrolases that degrade the extracellular matrix, could exacerbate neuronal injury after HIBI.^14^, ^15^ Evidence‐based, transcellular adhesion molecule 1 (NCAM‐1) and L1 cell adhesion molecule (L1CAM) in the cerebrospinal fluid (CSF) of six groups of neonates with HIE were counted, and it was concluded that L1CAM was elevated in posthemorrhagic hydrocephalus (PHH).^16^ Valdez et al. reported that serum PSA‐NCAM increased in mice after HI injury, and PSA‐NCAM had a better correlation with severity.^17^ On the other hand, the most significant KEGG pathways, pathways of neurodegeneration‐multiple diseases, were involved in oxidative stress, excitotoxicity, mitochondrial dysfunction, and autophagic damage from the perspective of cell‐autonomous mechanisms,^18^ which was parallel to HIE at the cellular level.^19^ Meanwhile, in the 20 most significant KEGG pathways, we also noted that the JAK‐STAT signaling pathway due to the STAT gene had the highest score of the critical nodes in PPI networks.

The STAT family consists of seven cytoplasmic transcription factors with extensive distributions and functions. It can also facilitate the pathophysiology, including the body's immune response, inflammatory response, and so on.^20^ Inflammation is a common event in HI, which can initiate the expression of STAT, nuclear factor‐κB (NF‐κB), and activator protein‐1 (AP‐1), which are regarded as proinflammatory genes regulating transcription factors.^21^ The JAK‐STAT pathway is considered as an important signaling mechanism for neuroprotective factors and mediates regeneration of nerves.^22^, ^23^ With the establishment of BV2 microglial cells under oxygen and glucose deprivation (OGD) and middle cerebral artery occlusion (MCAO) in mice as models of ischemia, the results from Xiang and his partners suggested that the ubiquitin‐specific protease 18 (USP18) reduced HI injury via the suppression of microglial activation by negatively inhibiting the JAK‐STAT pathway.^24^ Studies have indicated that the JAK‐STAT signaling pathway stimulated by upregulating miR‐183 could restrain apoptosis of hippocampal neurons and enhance neuronal proliferation in epilepsy rats.^25^ The downregulated miR‐28 expression can deactivate JAK‐STAT signaling pathways, which was predicted as a potential mechanism that lncRNA H19 参function to hypoxic neural‐like PC‐12 cells.^26^ Furthermore, studies have shown that overexpression of Hsp70 can interrupt STAT1, effectively downregulating the expression of proinflammatory genes in heat‐pretreated astrocytes to prevent brain ischemia through an anti‐inflammatory mechanism.^21^ There was a recent report that the STAT1 in the injured cortex of MCAO mice was upregulated. It also indicated that STAT1 could be dampened by overexpression of miR‐302a‐3p, and contributed to progression of ischemic brain injury.^27^ Additionally, Yin et al. found that salidroside could significantly inhibit the expression of IL‐6, TNF‐α, MCP‐1, STAT3, and NF‐κ‐B2 in an adult rat MCAO, and also inhibit cell apoptosis and reduce oxidative stress with downregulation of STAT3.^28^ A previous study has shown that JAK2/STAT3 signaling pathways serve an important function in the downstream signal pathway regulation of ischemic stroke‐related inflammatory neuronal damage. This experimental data showed that JAK2 and STAT3 phosphorylation increased in the ipsilateral cortex and striatum after transient middle cerebral artery occlusion in adult rats and 6–72 h of reperfusion. Compared to the vehicle control, in rats treated with AG490, as a JAK2 phosphorylation inhibitor, in the sham group, JAK2 and STAT3 phosphorylation did not occur after ischemia; also, the infarct volume and apoptotic cells were significantly decreased, and neurological deficits were improved. Similarly, the above results were also obtained after intracerebroventricular infusion of rats with siRNA (siRNA specific for STAT‐3 contributed to curtailed STAT‐3 mRNA expression and phosphorylation).^29^

Recently, Tian and his colleagues utilized real‐time quantitative reverse‐transcriptase polymerase chain reaction (qRT‐PCR) to test the JAK2‐related miRNA expression levels and western blot to analyze both OGD‐treated primary cultured neuronal cells and mouse brain with MCAO‐induced ischemic stroke. Bioinformatics showed that miR‐216a could be detected by 3ʹ untranslated region (3ʹUTR) dual‐luciferase assay to JAK2. Simultaneously, they carried out neurological deficit detection and neurological behavior grading to determine the infarction area and neurological deficits.^30^ Also, drugs such as fludarabine, a specific inhibitor of the STAT1 protein, exerted neuroprotective effects, whose latent mechanism could be mediated by weakening STAT1 phosphorylation and activating the cross‐regulation between STAT1 and STAT3 in neural cells.^31^ Moreover, immune cytokine IL‐23 (anti‐IL‐23) and the JAK3 inhibitor could protect from cerebral ischemia–reperfusion injury by targeting the immune‐specific JAK2‐STAT3 in the JAK/STAT pathway because they decreased infarct volume more effectively. Evidence suggested that phosphorylation levels of both JAK2 and STAT3 were augmented by anti‐IL‐23. Anti‐IL‐23 alleviated the injury by decreasing the levels of malondialdehyde (MDA) and superoxide dismutase (SOD) in the serum. Besides, the JAK3 inhibitor could strengthen the effect of anti‐IL‐23. In the above processes, the expression of JAK2 and STAT3 mRNA and protein were detected after anti‐IL‐23 treatment using a mouse model study, and the levels of MDA and SOD were detected by ELISA, ect.^32^ Collectively, all the data indicate that the exploration of the JAK/STAT pathway could lead to new therapies for neuronal recovery. We looking forward to further studies to evaluate the synergistic effect of pathways and related molecules.

An important finding of this study was that B2M, MRPS30, and VIM were upregulated in Paper 1, but contrasting results were obtained in Paper 2. Early studies confirmed that the urinary biomarker B2M was significantly higher in perinatal asphyxiated neonates than in controls when oliguria was present,^33^ and oliguria in the perinatal period is a sensitive indicator of infants at risk for long‐term neurologic deficits.^34^ Evidence also supported subclinical tubular dysfunction, probably secondary to hypoxic stress, combined with high levels of B2M in preterm infants.^35^ Not surprisingly, experimental data suggested that cardiomyocytes express and secrete elevated levels of B2M in response to ischemic damage, which could activate fibroblasts in a paracrine manner associated with therapeutic targets relevant for cardiac repair.^36^ In addition, Rist PM and his partners found that high levels of B2M were associated with an increased risk of ischemic stroke among women.^37^ However, MRPS30 could be weakly associated with HI injuries. To date, it has mainly been reported that its expression may significantly modulate breast cancer susceptibility, and encode a member of the mitochondrial ribosomal proteins to modulate mitochondrial activities.^38^ VIM is a wavy protein. It can encode the type III intermediate filament protein involved in the maintenance of cellular and tissue integrity. VIM is a major portion of the brain's cytoskeleton, with nestin and glial fibrillary acidic protein (GFAP) differentially expressed in terms of neurologic development.^39^, ^40^ The upregulation of VIM and GFAP is a cellular hallmark of reactive gliosis. Reactive astrocytes not only accelerate the formation of posttraumatic glial scars and inhibit central nervous system (CNS) regeneration but also compromise neural graft survival and integration, reduce the extent of synaptic regeneration, and inhibit regeneration of severed CNS axons.^41^ Katarina et al. came to the same conclusion, with reduced glial damage and glia scar formation and improved synaptic regeneration after nerve injury in adult mice lacking GFAP and VIM.^42^ Interestingly, upregulation of VIM expression is always accompanied by a higher‐level expression of STAT1 in the regulation of cellular immunity and apoptosis, invasion, and migration.^43^, ^44^, ^45^ Adopting a quantitative SUMO proteomics approach, VIM was identified as one of the protein inhibitors of activated STAT1 (PIAS1) substrates; PIAS1 is an E3 SUMO ligase. VIM sumoized cells migrate at higher levels than cells expressing non‐sumoized VIM mutants, indirectly predicting a role for PIAS1 and VIM in cell motility.^46^ More importantly, it was predicted that VIM may be the target of mir‐7a‐2‐3p by establishing an HI model in rats and an oxygen–glucose deprivation (OGD) model in vitro, and using bioinformatics and PCR to verify, suggesting that mir‐7a‐2‐3p plays a key role in HI injury, and is related to the regulation of VIM.^47^ Therefore, we speculate that the potential in‐depth mechanism of JAK/STAT signaling pathway‐mediated VIM has not been discovered.

Finally, the method described in this study can support an alternative pathway to learn about HIE, confirming its feasibility. Of course, it is difficult to guarantee the integrity of the data, but these data were obtained from the field of genomics and in terms of cellular molecular mechanisms with the gene network construction to improve work efficiency and reduce costs during the process of experimental research works, especially for subjects or samples that are not easy to collect, such as neonatal ischemic–hypoxic encephalopathy (NHIE) and genetic diseases in the embryo. In a word, we can benefit from it.

## 5 CONCLUSION

From the findings of SCS and bioinformatics predictive analytics model, our outcomes provided a better understanding of the roles of STAT, the JAK‐STAT signaling pathway, and VIM, which can provide an alternative avenue for further studies on HIE progression.

## CONFLICTS OF INTEREST

The authors declare no conflicts of interest.

## ETHICS STATEMENT

The ethics statement is not available.

## AUTHOR CONTRIBUTIONS

Hong‐Su Zhou completed and polished the manuscript. Ting‐Bao Chen came up with the idea of the review. All authors have no conflicts of interest to disclose. All authors have read and approved the final submitted manuscript.

## Supporting information

Supporting information.

Supporting information.

## Data Availability

Data have been deposited in GEO and DBGaP under accession number GEO: GSE67833, dbGaP: phs000989.v3. The data that support the findings of this study are openly available in GEO and DBGaP under accession number GEO: GSE67833, dbGaP: phs000989.v3. at doi:10.1016/j.stem.^7^; doi:10.1111/joa.12931.^9^
